# Relationships between negative life events and suicidal ideation among youth in China: The direct and moderating effects of offline and online social support from gender perspective

**DOI:** 10.3389/fpsyg.2022.998535

**Published:** 2022-10-06

**Authors:** Moye Xin, Julia Petrovic, Lijin Zhang, Xueyan Yang

**Affiliations:** ^1^School of Psychology, Shaanxi Normal University, Xi'an, China; ^2^Shaanxi Provincial Key Laboratory for Behavioral and Cognitive Psychology, Shaanxi Normal University, Xi'an, China; ^3^Shaanxi Provincial Key Research Center of Child Mental and Behavioral Health, Xi'an, Shaanxi, China; ^4^Human Development, McGill University, Montreal, QC, Canada; ^5^Institude for Population and Development Studies, School of Public Policy and Administration, Xi’an Jiaotong University, Xi'an, China

**Keywords:** offline social support, online social support, negative life events, suicidal ideation, youth, gender differences

## Abstract

**Background:**

Suicidal ideation was proved to be a critical precondition leading to the occurrence of subsequent suicidal behavior. Studies have confirmed that negative life events and forms of social support that youth are experiencing in the current socio-cultural context might have unique impacts on their suicidal ideation. However, the specific mechanism is relatively underexplored.

**Objective:**

We sought to investigate the impacts of offline and online social supports on Chinese students’ suicidal ideation under the pressure of various negative life events, as well as potential gender differences in these relationships.

**Methods:**

Participants were 2,018 middle – high school and university students from Northwestern China, who completed a demographics questionnaire and self-report measures of negative life events, social support, and suicidal ideation.

**Results:**

Offline social support had a significant direct effect on suicidal ideation across genders. Among male youth, offline social support only had a moderating effect on the relationship between punitive negative life events and suicidal ideation. Among female youth, offline social support had a significant moderating effect on suicidal ideation under the pressure of all types of negative life events; Online social support only had a significant direct effect on female youth’s suicidal ideation, although it did significantly moderate the relationship between all types of negative life events and suicidal ideation, across genders.

**Conclusion:**

Our findings revealed direct and moderating effects of offline and online social support on suicidal ideation among youth under the pressure of different types of negative life events, as well as gender-specific patterns in these relationships.

## Introduction

### Suicidal ideation among youth

Globally, suicidal thoughts and behaviors have seriously endangered the physical and mental health of youth, with far-reaching consequences that impact their families, schools, and communities (Ertl et al., 2020; Lyu et al., 2021). Classic studies have found that the emergence of suicidal behavior is not achieved overnight; rather, there is an underlying psychological and behavioral process that precedes it (Roy, 1983; Gould et al., 2003, 2004), which often includes 3 necessary stages: suicidal ideation, suicidal plan, and suicidal action (Heeringen et al., 2011). Relevant studies have found that suicidal ideation was clearly expressed prior to about two thirds of suicidal casualties (Yoder and Hoyt, 2008), providing evidence that suicidal ideation is an important precondition leading to the occurrence of subsequent suicidal behavior (Williford et al., 2021). In fact, suicidal ideation is the most important index in the suicide risk assessment conducted by the World Health Organization (Castillo et al., 2020). According to a study of Chinese middle school students, 5% of students who had experienced suicidal ideation subsequently engaged in NSSI (Non-suicidal self injury) or even actual suicidal behavior afterwards, suggesting that suicidal ideation may be predictive of NSSI and subsequent suicidal behavior among Chinese youth (Sun et al., 2006; Ruan et al., 2022).

The study of suicidal ideation and its associated factors is conducive to screening at-risk groups and taking effective prevention and intervention measures. Sociologists have repeatedly emphasized that research cannot separate individuals from the social environment in which suicide occurs (Pompili et al., 2005; Borges et al., 2008). Accordingly, social psychologists have taken a more comprehensive approach to suicide research that takes into account the socio-cultural environment of specific historical periods (e.g., information era; post-COVID era), which may have far-reaching impacts on suicidal behavior (Bao et al., 2019; Roberts and Chen, 2019; Sedgwick et al., 2019; Bailey et al., 2020; Daou et al., 2021). Therefore, we propose a general hypothesis that the different types of negative life events and forms of social support that youth are experiencing in the current socio-cultural context may have unique impacts on their suicidal ideation.

### Negative life events as a risk factor

Relevant research has suggested that negative life events, defined as unpleasant events that have occurred in one’s living environment and which bring about negative psychological, physiological, and behavioral consequences (e.g., failure in an exam, experiencing theft, losing a loved one, etc; Kendler et al., 2001), may be related to the occurrence of suicidal ideation and subsequent behaviors. Negative life events are a particularly robust risk factor for suicidal ideation (Gould et al., 2003). The research on the relationship between negative life events and suicidal ideation has gradually developed into a research hotspot in sociology, psychology, and other related disciplines (Roberts and Chen, 2019; Stewart et al., 2019; Tasmim et al., 2020). Notably, the risk for suicidal ideation is significantly higher among individuals who encounter intense pressures or negative events in their daily life, relative to the general population (Mcauliffe et al., 2003). Moreover, a Chinese study found that negative life events were significantly predictive of suicidal ideation, whereby the more negative life events an individual encounters, the greater the intensity of one’s suicidal ideation would crystallize (Huang et al., 2007). Relevant research has also shown that the cumulative effect of negative life event pressures might lead to the emergence of other adverse psychological symptoms such as depression, paranoia, and hostility, and that these psychological symptoms may subsequently aggravate the intensity of suicidal ideation (Muscatell et al., 2009; Jiao et al., 2010; Thompson et al., 2012). Additionally, negative life events may lead to non-suicidal self-injury (NSSI; Fu et al., 2017; Yu et al., 2019), and NSSI has been found to be a risk factor for suicidal ideation and suicide plan (Nock et al., 2006). Taken together, the above studies demonstrate that negative life events are inextricably linked to suicidal ideation, such that they are one of the most critical risk factors for suicidal ideation. However, research has not yet investigated whether different types of social support (e.g., offline or online) may differentially impact the relationship between negative life events and suicidal ideation among youth; such an investigation is needed to further clarify micro-level mechanisms that may predict suicide risk.

### Social support as a protective factor

Social support is a multi-dimensional concept, which includes both cognitive elements and environmental factors (Feldman and Cohen, 2000). Cognitively speaking, the relevant research divides social support into two types due to different subjective and objective feelings of the subject (Manuel, 1986). The first type is the support that really exists in the objective world without human will. As the object of real existence, such support does not change due to the influence of personal perception (e.g., government aid and subsidy, social welfare, etc); The second type refers to emotional support that can be perceived by individuals at the subjective level, which mainly lies in the perception process that individuals are respected, understood and supported in social activities, with the subjective satisfaction of such support (Dunkel-Schetter and Bennett, 1990). This kind of support is also called Perceived Social Support (PSS), which is closely related to the subjective feelings of individuals.

Environmentally speaking, this study refers to the research of Chinese scholar Liang, who divided social support into two new categories: offline and online social support (Liang and Liu, 2010). The premise for the establishment of this classification is that, based on various social interpersonal communication as a platform, social support can have the attribute of giving and receiving, and its essence lies in the interaction and exchange of human beings as social animals. “Life without supporting relationship in modern society is difficult to exist” (Albrecht and Goldsmith, 2003). The platforms used to exchange social support gradually change in alignment with technological advances (Bruhn, 2011). After the new millennium, internet-based social communication has grown in popularity and is increasingly being used for exchanges of social support globally, through emotional online communication which might foster a sense of belonging among those taking part. The rapid development of technology has led to an advancement in possibilities for more diverse communications, which might affect individuals’ levels of perceived social support, especially among adolescents, youth and young adults (Suzuki et al., 2020). Internet-based social communication has grown in popularity and is increasingly being used for exchanges of social support, through emotional online communication which may foster a sense of belonging among those taking part (Utz and Breuer, 2017). Social communication activities based on the virtual world also show that members not only exchange information with each other, but also exchange social support with each other, such as emotional online communication or sense of belonging (Ridings et al., 2018). In addition, traditional research on social support is defined based on the actual situation of the real society, and there is not much involved on the internet and its related factors. Therefore, the academic community tends to call the previous one offline social support, and regard the latter one formed by the support of the Internet platform as the premise of the existence of online social support. Therefore, it is also reasonable to divide social support into offline (i.e., support that is exchanged in-person) and online social support (i.e., support that is exchanged over the Internet) according to different environments (Liang and Liu, 2010).

Studies have suggested that offline social support may be a protective factor for adolescents’ mental health and adaptability (Brissette et al., 2002; Levendosky et al., 2002; Gx et al., 2020). According to Liang and Liu (2010), offline social support may be described as distinct from online social support, wherein individuals subjectively feel the emotional experience of being respected, understood, and supported in real-life social contexts.

Notwithstanding the natural attributes of offline social support, the rapid development of technology has led to an advancement in possibilities for online communication, which has evolved to a new virtual society with greater efficiency, a wider audience, more advanced interaction technology, greater impact, and more diverse functions, all of which may affect individuals’ daily lives and levels of perceived social support in important ways (Suzuki et al., 2020). Thus, the present study defines online social support as the sense of identity and belonging obtained when one feels understood and respected in the context of emotional exchanges conducted through virtual platforms.

Distinct from offline social support, online social support can not only help improve the health of individuals troubled by stressful events, but also have implications for the prevention of life-threatening behaviors such as self-injury and suicide (Whitlock et al., 2006; Williams, 2013). Furthermore, a noteworthy study by Idriss et al. (2009) reported improved quality of life, perceived social support, and physical health symptoms among participants who engaged in online support groups by posting messages, replying to messages, and searching for information within them. Thus, seeking online social support may generate more positive emotions, alleviate pressures and stress from challenging life circumstances, and thereby reduce the likelihood of resorting to health risk behaviors.

However, research findings on the potential benefits of online social support are mixed, with some studies suggesting that the impact of online social support on mental health is not always positive (Yao et al., 2021; Zheng et al., 2021). Specifically, given the potentially transient nature of online support, the sense of respect and support brought about by virtual communications may not alleviate life pressures in a sustained manner (Holmstrom and Burleson, 2015). Additionally, repeated reliance on online support groups may lead to Internet addiction, which is characterized by excessive or poorly controlled preoccupations, urges or behaviours regarding computer use and internet access, that might lead to impairment or distress (Shaw and Black, 2015). Even online social relationships may appear as substitutes for in-person social relations, particularly when individuals do not feel recognized or satisfied in their day-to-day life (Tseng and Yang, 2015). Online social groups may also bring about other hidden dangers, such as exposure to social support from antisocial personality online groups, which may provoke health risk behaviors in those taking part (Cummins and Lau, 2010). Therefore, the effect of online social support on the relationship between negative life events and suicidal ideation among youth needs further clarification.

### Research gaps and objectives

Few Chinese studies have assessed the relationship between negative life events, suicidal ideation, and social support among youth, and most of them employed cross-sectional descriptive analyses (Xu, 2010; Yang et al., 2015; Liu et al., 2019). In terms of offline social support, studies have explored the impact of offline social support on the relationship between negative life events and suicidal ideation among youth, and found that it played a mediating role (Liu and Xiao, 2002). Offline social support has also been found to have a moderating effect on the relationship between negative life events and suicidal ideation (Liu, 2017). However, there is a lack of research on the potential moderating effect of online social support on the relationship between negative life events and suicidal ideation among youth.

Thus, this paper addressed the aforementioned gaps in the literature, as follows: (1) As the most mainstream and well-documented form of social support, offline social support has real-time convenience and directness that online social support does not; as such, offline social support can have a far-reaching impacts on individual health behavior (Strand et al., 2020). However, it remains unclear whether offline social support might inhibit or protect suicidal ideation among youth of different genders under the pressure of different types of negative life events. Relevant research has shown that offline social support may backfire or even aggravate health risk behavior when it surpasses a certain threshold (Li et al., 2019). Moreover, given that negative life events represent a multi-dimensional concept, the potential role of offline social support in the relationship between different types of negative life events and suicidal ideation remains unclear.

(2) The role of online social support on suicidal ideation among youth under the pressure of negative life events is another noteworthy issue that researchers have been paying increasing attention to. Studies have shown that online social support may have differential impacts on the relationship between negative life events and suicidal ideation as a function of the population of interest (Tao and Zheng, 1999). For instance, online social support has been found to be very helpful among patients with Huntington disease (HD) as it plays a role in alleviating anxiety, managing stress, and reducing health risk behaviors (Divino et al., 2012). However, it remains to be determined whether online social support may play a similar stress-buffering role among youth more generally. While we suggest that online social support impacts the relationship between negative life events and suicidal ideation among youth, whether this impact is protective or aggravating remains unknown.

**Figure 1:**
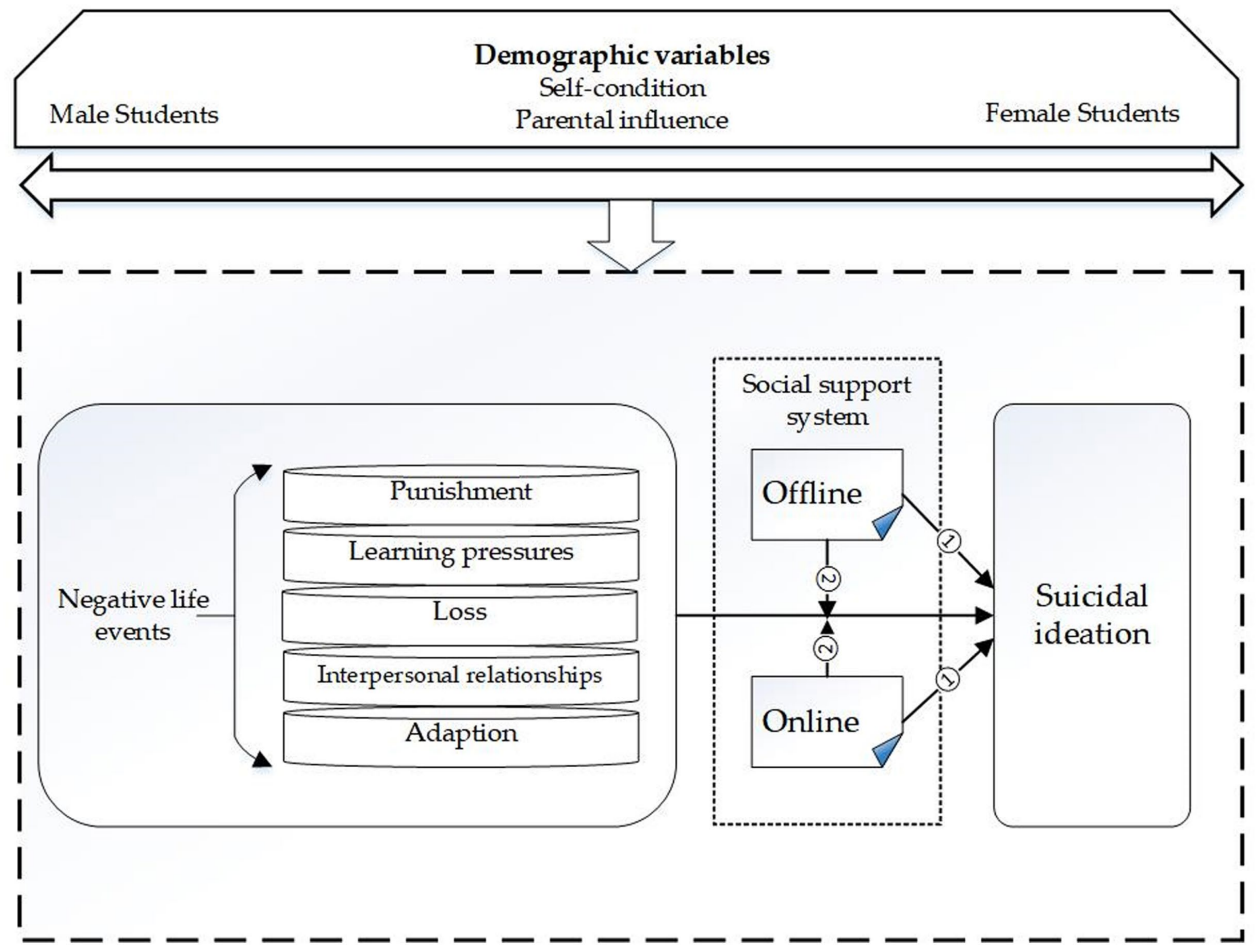
The direct and moderating effects of offline and online social support on suicidal ideation among youth under the pressure of negative life events (Path 1–2).

The present study thus assessed two main research objectives. The first objective was to determine whether offline (Objective 1A) and online (Objective 1B) social support had direct effects on suicidal ideation among youth, across genders (see Figure 1; Path 1). The second objective was to determine whether offline (Objective 2A) and online (Objective 2B) social support significantly moderated the relationship between negative life events and suicidal ideation among youth, across genders (see Figure 1, Path 2). It was hypothesized that offline (H1A) and online (H1B) social support would both have significant direct effects on suicidal ideation among youth, across genders. In addition, offline (H2A) and online (H2B) social support were expected to significantly moderate the relationship between negative life events and suicidal ideation among youth, across genders.

## Materials and methods

### Participants

Participants were a sample of 2,018 middle school and university students (803 males, 1,215 females; 908 middle school students, 1,110 university students; *M*_age_ = 17.8 years; age range: 11–25 years) recruited from six high schools and three universities in Northwestern China, individuals with mental impairment or neurological conditions were not included in this survey, participants can withdraw at any time if they feel uncomfortable during the survey. Parents or legal guardians gave permission for high school students’ participation by signing consent forms after receiving the paper version of ethical review. The study was approved by the institutional ethics review board within the university and the schools where the research was conducted.

### Measures

#### Suicidal ideation

The Scale for Suicide Ideation (SSI) was used to assess suicidal ideation (Beck et al., 1979). This measure consists of 14 items (e.g., *I think suicide can end the current pain*; *I have taken some strange or dangerous drugs to suicide on purpose*) rated on a 5-point Likert scale, ranging from 1 (*completely disagree*) to 5 (*completely agree*). Higher scores indicate greater suicidal ideation intensity. The SSI demonstrated good reliability in the present study (α = 0.81).

#### Negative life events

The Adolescent Self-Rating Life Events Checklist (ASLEC) was used to examine negative life events (Liu et al., 1997). This scale consists of 27 items, each categorized within 1 of 6 types of negative life events: interpersonal relationships (e.g., *was misunderstood or wronged; was discriminated against or treated coldly*; *α* = 0.83), learning pressures (e.g., *failure in an exam*; α = 0.88), punishment (e.g., *was criticized or punished at school*; α = 0.91), loss (e.g., *sudden death of relative or friend; experienced theft or lost items*; α = 0.84), adaptation (e.g., *transfer or suspension, major changes in daily routines*; α = 0.89), and other (e.g., *family financial problems*; α = 0.87). Respondents rated each item on a 5-point Likert scale based on the extent to which each event impacted their life, from 1 (*no impact*) to 5 (*extremely severe impact*). A total of 5 factors were extracted; this 5-factor model met the numerical requirements of the fitting index test without changing the original 6-factor connotation of the original scale, and the fitting index result was more ideal than the 6-factor model. The correlation coefficient of the 5-factor model ranged from 0.69 to 0.79 (*p* = 0.006) and the structure validity was improved. In terms of structure and content, compared with the original 6-factor model, the 5-factor model omits the “other types” factor. Therefore, in the present study, this scale assessed 5 types of negative life events among youth: interpersonal relationships, learning pressures, punishment, loss, and adaptation. In the current study, the modified ASLEC demonstrated good reliability (*α* = 0.87).

#### Offline social support

A modified version of the Multidimensional Scale of Perceived Social Support (MSPSS) was used to assess offline social support (Zimet et al., 1990; Xiao, 1994). The original measure includes 12 items which divide into 3 subscales related to the source of support, namely family, friends, or significant other. This scale was adapted for Chinese contexts by Xiao (1994) (e.g., *When I have problems, my family will show up beside me; I can rely on my friends in times of difficulty, etc*), in order to facilitate understanding and cultural division, by modifying the original subscales (family, friends, significant other) to teachers, friends, and family members. The adapted scale consists of 16 items scored on a 5-point Likert scale ranging from “completely inconsistent” to “fully consistent.” The total score for offline social support is obtained by calculating the mean of item responses, where higher mean scores indicate higher levels of perceived social support. The modified scale showed good reliability in the present study (*α* = 0.904).

#### Online social support

The 23-item online social support scale compiled by Liang and Liu was used (Liang and Liu, 2010) to assess the degree to which participants have obtained emotional and practical support through the Internet. Participants are asked to indicate the extent to which they have received online social support using a 5-point Likert scale ranging from 1 (no) to 5 (always). Sample items include, “When I feel lonely, I talk with others through the Internet,” and, “I receive life advice from others through online communications.” A mean score is calculated whereby the higher the score, the more online social support received. The scale demonstrated good reliability in the present study (*α* = 0.835).

#### Demographic variables

In order to support the reliability of the regression analysis, this paper also included four demographic variables. The specific measurements are as follows: (1) Age. Continuous variable. The subjects were measured from the lowest age 11 to the highest age 25; (2) School stages. Categorical variable. The measurement is divided into two categories: “0 = middle school, 1 = university”; (3) Single child or not. Categorical variable. “0 = single child, 1 = not single” for measurement. (4) Parents’ marital status. Categorical variable. The measurement is divided into four categories: “1 = first marriage, 2 = remarriage, 3 = divorce, 4 = widowed.”

### Data collection

The present study used convenience sampling to recruit teachers within six middle schools and three universities who expressed interest in having their students participate. Within the schools, stratified sampling was used to recruit participants, in order to ensure equal representation across genders and levels of study within the middle schools (i.e., from grade 8 to grade 12) and universities (i.e., from first to fourth year). All questionnaires were anonymous. A total of 2,400 questionnaires were distributed and 2,018 valid questionnaires were recovered after 7 days of investigating (if participants took questionnaires back with them at home, they were asked to bring back the questionnaire the next day ignoring the completion). The participation rate in the present study was therefore 84.08%.

### Data analysis strategy

A series of one-way ANOVAs were conducted to analyze potential gender differences in negative life events, suicidal ideation and social support amongst youth.

Furthermore, the ordinary least squares (OLS) linear regression method was employed to test the direct and moderating effects of offline and online social support on suicidal ideation among youth under the pressure of negative life events, across genders.

The present model was initially proposed by Cohen (2004) to determine whether social support may have a “pressure-buffering effect” in the face of significant life stress. In this study, the potential moderating effects of offline and online social support were assessed by exploring whether the relationship between negative life events and suicidal ideation would change with the intervention of offline and online social support.

6 models were thus assessed; see Table 1 for details regarding each model (O = offline, N = online, M = male, F = female). Model 1 (M/F) included offline social support (OSS) and online social support (NSS) as an independent variable to measure its main effect on the relationship between negative life events (independent variable) and suicidal ideation (dependent variable). Subsequently, on the basis of Model 1, the interaction terms for punishment, learning pressures, loss, interpersonal relationships, and adaptation as different types of negative life events were successively included in Models 2–6 to determine the potential moderating effects of offline and online social support on suicidal ideation.

**Table 1:** Relevant models’ information.

Dependent variables	Model	Independent variables
Suicidal ideation intensity among males	Model O (N) 1M	Negative Life Events + OSS + NSS + Demographic variables(Age, only child or not, parents’ marital status)
	Model O (N) 2M	Negative Life Events + OSS + NSS + Punishments * OSS (NSS) + Demographic variables
	Model O (N) 3M	Negative Life Events + OSS + NSS + Learning pressures * OSS (NSS) + Demographic variables
	Model O (N) 4M	Negative Life Events + OSS + NSS + Loss * OSS (NSS) + Demographic variables
	Model O (N) 5M	Negative Life Events + OSS + NSS + Interpersonal relationship * OSS (NSS) + Demographic variables
	Model O (N) 6M	Negative Life Events + OSS + NSS + Adaption * OSS (NSS) + Demographic variables
Suicidal ideation intensity among females	Model O (N) 1F – 6F	Same procedure as above

M stands for “males,” F for “females,” O for “offline,” N for “online,” OSS for “offline social support,” NSS for “online social support.”

## Results

### Descriptive analysis results

Among the 2018 participants, 127 students demonstrated relatively intense suicidal ideation, accounting for 6.3% of the total, including 82 middle school students, 45 university students, 71 males and 56 females. Results from one-way ANOVAs revealed significant gender differences in suicidal ideation intensity (*F* = 2.12, *p* < 0.0001) and overall reports of negative life events (*F* = 9.198, *p* < 0.001), whereby males reported higher levels of both relative to females (see Table 2 for means and standard deviations). Moreover, analyses of gender differences across the 5 types of negative life events revealed that males reported a significantly higher degree of negative life events related to punishment (*F* = 6.083, *p* < 0.001), learning pressures (*F* = 2.777, *p < 0.1*), loss (*F* = 5.277, *p < 0.05*), and interpersonal relationships (*F* = 9.924, *p < 0.01*). No significant gender differences emerged in reports of negative life events related to adaptation (*F* = 1.135, *p* = 1.651). No significant gender differences emerged in reports of offline social support (*F* = 0.412, *p* = 3.318). Additionally, analyses of online social support revealed that males received more social support from the Internet than females (*F* = 12.638, *p* < 0.001). Refer to Table 2 for detailed information regarding all descriptive analyses.

**Table 2:** Descriptive analysis.

	Male (*N* = 803)		Female (*N* = 1215)	
	Min/Max	Mean(SD)	Min/Max	Mean(SD)
Suicidal ideation intensity	1/5	1.53(0.88)	1/5	1.41(0.68)
	*F* = 2.12^***^			
Negative life events (Overall)	1/5	1.79(0.65)	1/5	1.67(0.57)
	*F* = 9.198^***^			
Punishments	1/5	1.60(0.71)	1/5	1.36(0.51)
	*F* = 6.803^***^			
Study stress	1/5	2.24(0.77)	1/5	2.18(0.69)
	*F* = 2.777^+^			
Losing	1/5	1.63(0.62)	1/5	1.58(0.54)
	*F* = 5.277^*^			
Interpersonal relationship	1/5	2.18(0.74)	1/5	2.08(0.65)
	*F* = 9.924^**^			
Adaption	1/5	1.85(0.71)	1/5	1.82(0.59)
	*F* = 1.135			
Offline social support (Overall)	1/5	3.38(0.82)	1/5	3.35(0.73)
	*F* = 0.412			
Online social support (Overall)	1/5	2.71(0.73)	1/5	2.31(0.65)
	*F* = 12.638^***^			

+ *p* < 0.1;

* *p* < 0.05;

** *p* < 0.01; and

*** *p* < 0.001.

### The direct and moderating effects of offline social support

As demonstrated in Table 3 (Model O1M), results revealed that offline social support had a significant negative direct effect on suicidal ideation among male youth (−0.125, *p < 0.001*). In other words, the more offline social support males reported receiving, the less intense their reported suicidal ideation was.

**Table 3:** The direct and moderating effects of offline social support on suicidal ideation among male and female youth.

Independent Variables	Suicidal ideation intensity for males						Suicidal ideation intensity for females					
	Model O1M	Model O2M	Model O3M	Model O4M	Model O5M	Model O6M	Model O1F	Model O2F	Model O3F	Model O4F	Model O5F	Model O6F
*Negative life events*												
Punishment	0.204^***^	0.190^***^	0.199^***^	0.199^***^	0.198^***^	0.204^***^	0.048	0.057^***^	0.052	0.060	0.047	0.056
Learning pressures	0.008	0.014	0.154	0.012	0.014	0.008	0.099^**^	0.105^**^	0.785^***^	0.108^***^	0.104^***^	0.109^***^
Loss	0.001	0.001	−0.001	0.001	0.001	0.001	0.065^+^	0.084^+^	0.074^+^	0.071^+^	0.075^+^	0.065^+^
Interpersonal relationships	0.001	0.006	0.006	0.005	0.150	0.001	0.119^***^	0.124^***^	0.120^***^	0.130^***^	0.616^***^	0.131^***^
Adaptation	0.120^+^	0.116^+^	0.117^+^	0.119^+^	0.114^+^	0.116^+^	0.101^**^	0.097^+^	0.096^+^	0.091^+^	0.102^+^	0.566^***^
*Social support system*												
Offline social support	−0.125^***^	−0.277^***^	−0.229^***^	−0.383^***^	−0.388^***^	−0.383^***^	−0.114^***^	−0.181^***^	−0.235^***^	−0.238^***^	−0.236^***^	−0.217^***^
Online social support	0.050	0.047	0.051	0.050	0.043	0.051	0.054^*^	0.051^*^	0.051^*^	0.053^*^	0.050^*^	0.051^*^
*Control variables*												
Age	0.051	0.059	0.054	0.055	0.055	0.051	−0.051^***^	−0.147^***^	−0.147^***^	−0.145^***^	−0.148^***^	−0.136^***^
Schooling stages: University (Middle school)	−0.032	−0.029	−0.031	−0.033	−0.031	−0.031	−0.007	−0.009	−0.007	−0.004	−0.007	−0.007
Single child (not single)	−0.011	−0.012	−0.011	−0.012	−0.012	−0.011	−0.011	0.013	0.013	0.012	0.011	0.017
Marital status of parents: Remarriage (first marriage)	0.058^+^	0.060^+^	0.059^+^	0.059^+^	0.059^+^	0.058^+^	0.058	0.012	0.012	0.007	0.013	0.011
Divorced (first marriage)	0.083^+^	0.083^+^	0.083^+^	0.083^+^	0.082^+^	0.083^+^	0.083	0.011	0.006	0.003	0.014	0.008
Widowed (first marriage)	0.049	0.048	0.048	0.049	0.048	0.049	0.049	0.026	0.026	0.030	0.025	0.028
*Moderation analysis*												
Punishment * Offline social support		−0.203^*^						−0.543^***^				
Learning pressures * Offline social support			−0.157						−0.754^***^			
Loss * Offline social support				−0.134						−0.585^***^		
Interpersonal relationships * Offline social support					−0.161						−0.536^***^	
Adaptation * Offline social support						0.004						−0.505^***^
df	11	11	11	11	11	11	12	12	12	12	12	12
Adjusted *R*^2^	0.156	0.157	0.157	0.159	0.162	0.168	0.225	0.242	0.250	0.247	0.258	0.234
*F*	14.496^***^	14.557^***^	15.412^***^	16.529^***^	18.126^***^	18.646^***^	44.598^***^	42.323^***^	44.096^***^	43.529^***^	45.126^***^	40.646^***^
Observations	802	802	802	802	802	802	1214	1214	1214	1214	1214	1214

M stands for “males,” F for “females,” O for “offline social support.” All regression coefficients reported in the table were standardized regression coefficients.

+ *p* < 0.10,

* *p* < 0.05;

** *p* < 0.01 and

*** *p* < 0.001.

With the addition of interaction terms (i.e., Models O2M-O6M), it was revealed that offline social support had a significant and negative moderating effect on the relationship between punishment-related negative life events and suicidal ideation among males (−0.203, *p* = 0.027). Specifically, offline social support decreased the positive effect of punishment-related negative life events on male youth’s suicidal ideation. Offline social support did not have a moderating effect on the relationship between other types of negative life events and suicidal ideation among male youth.

Among female youth, offline social support also had a significant negative direct effect on their suicidal ideation (Model O1F; −0.114, *p* < 0.001). That is, the more offline social support females reported receiving, the less intense their reported suicidal ideation was, consistent with our findings among male youth.

However, with the addition of interaction terms (i.e., Models O2F-O6F), it was found that offline social support had a significant and negative moderating effect on the relationship between all types of negative life events and suicidal ideation among females (−0.543, −0.754, −0.585, −0.536, −0.505, all *p*’s < 0.*001*). In other words, offline social support decreased the positive effect of all types of negative life event on female youth’s suicidal ideation, a finding that was distinct from those of our male participants.

### The direct and moderating effects of online social support

As demonstrated in Table 4 (Model N1M), results revealed that online social support did not have a significant direct effect on suicidal ideation among male youth (0.050*, p* = 0.362). In other words, the amount of online social support received by male students was not directly related to the intensity of their suicidal ideation. However, with the addition of interaction terms (Models N2M-N6M), it was found that online social support had a significant negative moderating effect on the relationship between all types of negative life events and suicidal ideation among male youth (−0.241, −0.218, − 0.206, − 0.214, −0.178, all *p*’s < 0.001). Thus, online social support decreased the impact of all types of negative life events on male students’ suicidal ideation.

**Table 4:** The direct and moderating effects of online social support on suicidal ideation among male and female youth.

Independent variables	Suicidal ideation intensity for males						Suicidal ideation intensity for females					
	Model N1M	Model N2M	Model N3M	Model N4M	Model N5M	Model N6M	Model N1F	Model N2F	Model N3F	Model N4F	Model N5F	Model N6F
*Negative life events*												
Punishment	0.204^***^	0.421^***^	0.193^***^	0.193^***^	0.192^***^	0.190^***^	0.048	0.299^***^	0.048	0.050	0.044	0.049
Learning pressures	0.008	0.019	0.213^***^	0.017	0.019	0.016	0.099^**^	0.101^**^	0.360^***^	0.101^***^	0.100^***^	0.102^***^
Loss	0.001	0.001	0.003	0.194^***^	0.002	0.002	0.065^+^	0.073^+^	0.066^+^	0.302^***^	0.069^+^	0.064^+^
Interpersonal relationships	0.001	0.002	0.004	0.003	0.195^***^	0.003	0.119^***^	0.112^***^	0.106^***^	0.113^***^	0.344^***^	0.114^***^
Adaptation	0.120^+^	0.108^+^	0.107^+^	0.112^+^	0.105^+^	0.286^***^	0.101^**^	0.086^**^	0.083^+^	0.083^+^	0.087^+^	0.320^***^
*Social support*												
Offline social support	−0.125^***^	−0.118^***^	−0.121^***^	−0.123^***^	−0.127^***^	−0.211^***^	−0.114^***^	−0.121^***^	−0.126^***^	−0.110^***^	−0.114^***^	−0.119^***^
Online social support	0.050	−0.044	−0.045	−0.044	−0.388	−0.037	0.054^*^	0.060^*^	0.235^***^	0.061^**^	0.063^*^	0.064^**^
*Control variables*												
Age	0.075	0.049	0.043	0.046	0.045	0.052	−0.051^***^	−0.174^***^	−0.180^***^	−0.175^***^	−0.177^***^	−0.170^***^
Schooling stages: University(Middle school)	−0.007	−0.009	−0.007	−0.004	−0.007	−0.007	−0.007	−0.029	−0.031	−0.033	−0.031	−0.031
Single child (not single)	−0.013	−0.015	−0.014	−0.015	−0.015	−0.014	−0.011	0.010	0.011	0.009	0.009	0.012
Marital status of parents: Remarriage (first marriage)	0.063^+^	0.058^+^	0.058^+^	0.058^+^	0.057^+^	0.059^+^	0.058	0.012	0.012	0.010	0.012	0.012
Divorced (first marriage)	0.083^*^	0.083^*^	0.083^*^	0.083^+^	0.082^+^	0.084^+^	0.083	0.010	0.008	0.007	0.012	0.009
Widowed (first marriage)	0.042	0.050	0.049	0.050	0.050	0.049	0.049	0.029	0.030	0.031	0.029	0.030
*Moderation analysis*												
Punishment * Online social support		−0.241^***^						−0.295^***^				
Learning pressures * Online social support			−0.218^***^						−0.290^***^			
Loss * Online social support				−0.206^***^						−0.260^***^		
Interpersonal relationships * Online social support					−0.214^***^						−0.255^***^	
Adaptation * Online social support						−0.178^***^						−0.253^***^
df	11	11	11	11	11	11	12	12	12	12	12	12
Adjusted *R*^2^	0.142	0.154	0.157	0.158	0.161	0.162	0.148	0.152	0.154	0.158	0.161	0.162
*F*	13.116^***^	14.557^***^	14.412^***^	14.529^***^	14.126^***^	14.646^***^	20.136^***^	22.323^***^	22.096^***^	21.429^***^	23.156^***^	22.647^***^
Observations	802	802	802	802	802	802	1214	1214	1214	1214	1214	1214

M stands for “males,” F for “females,” N for “online social support.” All regression coefficients reported in the table were standard regression coefficients.

+ *p* < 0.10,

* *p* < 0.05;

** *p* < 0.01; and

*** *p* < 0.001.

As demonstrated in Table 4 (Model N1F), results revealed that online social support had a significant and positive direct effect on suicidal ideation among female youth as well (0.054*, p* = 0.046). In other words, the more online social support females received, the higher the intensity of suicidal ideation they might have.

Furthermore, with the addition of interaction terms (Models N2F-N6F), it was found that online social support had a significant negative moderating effect on the relationship between all types of negative life events and suicidal ideation among female youth (−0.295, −0.290, −0.260, −0.255, −0.253, all *p*’s < 0.001). In other words, online social support weakened the relationship between all types of negative life events and suicidal ideation among female students, which was similar to our findings from the male students.

## Discussion

### The direct effect of offline social support among males

As hypothesized (H1A), offline social support had a significant negative direct effect on suicidal ideation among male youth in the present study. This finding suggests that the more offline social support male youth perceived receiving, the less intense their reported suicidal ideation was. This finding is consistent with previous studies which have found that the acquisition of offline social support may significantly reduce the intensity of suicidal ideation (Xiao, 1994; Yan and Zheng, 2006; Li, 2011). With adequate offline social support, adolescents and young adults may be better equipped to cope with challenges in their lives, maintain their physical and mental health, and reduce the intensity of any suicidal ideation they may experience (O'Hara, 2006).

### The moderating effect of offline social support among males

Also as hypothesized (H2A), offline social support was found to exert a moderating effect on the relationship between punishment and suicidal ideation among male youth. Chinese gender studies have found male adolescents and young adults to be naturally lively, rebellious, and difficult to discipline; thus, they report more frequent punishment relative to females and are more vulnerable to the negative impacts of punishment (Lai, 2006; Zhao, 2007). It is therefore possible that male youth may seek offline social support in response to their experiences of punitive negative life events to alleviate their psychological distress, thereby reducing the intensity of suicidal ideation which can be brought about by an accumulation of negative emotions (Su et al., 2006). Nevertheless, contrary to H2A, offline social support had no moderating effect on the relationship between suicidal ideation and any other type of negative life events.

### The direct effect of offline social support among females

As hypothesized (H1A), offline social support also had a significant negative direct effect on the suicidal ideation of female youth, such that the more offline social support female youth perceived, the lower the intensity of their suicidal ideation. This finding is also consistent with previous studies which have found that the acquisition of offline social support may significantly reduce the intensity of suicidal ideation (Xiao, 1994; Yan and Zheng, 2006; Li, 2011), given the gender differences, the possible explanation could be that, compared with male students, female students’ perception of shame and alexithymia is more obvious, which leads to women’s tendency to use offline social support to talk to their close friends or family members when facing different types of negative life event pressures, thus causing suicidal ideation, in order to solve their own bad emotions and get rid of the trouble of suicidal psychology (Feng et al., 2016).

### The moderating effect of offline social support among females

Contrary to our findings from male youth but consistent with our hypothesis (H2A), offline social support had significant negative moderating effect on the relationship between all types of negative life events and suicidal ideation among female youth, with the strongest effect on the relationship between learning pressures and suicidal ideation. This finding is consistent with Chinese gender studies, which have reported that female students in China demonstrated greater institutional compliance and psychological vulnerability relative to male students, due to traditional gender traits (Zhou, 2019). Therefore, learning pressures may be particularly salient among female students, who are likely to experience marked distress in response to them as a result of coping difficulties (Yao, 2018), and may experience greater suicidal ideation as a result of this (Liu, 2010; Chen, 2016). Offline social support provided by family, friends, or educators may thus be central to alleviating distress associated with learning pressures among female youth which can, in turn, decrease the potential for intense suicidal ideation (Wang, 2016).

Furthermore, the significant negative moderating effect of offline social support on the relationship between negative life events associated with interpersonal relationships and female students’ suicidal ideation may be explained by female Chinese students’ sensitivity to various emotions. Specifically, in the face of interpersonal conflicts, female youth have been found to have a relatively lower ability to rationalize such conflicts compared to male youth (Zhang et al., 2011), which may place them at-risk for marked distress stemming from interpersonal relationships and subsequent suicidal ideation. Thus, offline social support may alleviate the negative impacts of interpersonal challenges among female youth, thus reducing the likelihood that these challenges may lead to suicidal ideation (Li, 2018).

Previous Chinese studies have found that personal loss may naturally result in greater distress among female youth, relative to male youth, due to traditional gender traits (Zhou, 2019). Thus, effective offline social support may foster their psychological resilience and ability to cope with experiences of loss (Lu and Ding, 2009). Therefore, offline social support may be an important protective factor in the relationship between negative life events related to personal loss (e.g., of a family member or loved one) and suicidal ideation among female youth.

Negative life events related to adaptation may also place female youth at-risk of suicidal ideation (Shi, 2012). Relevant studies have found that among female students, social adaptation may be effective factors in predicting suicide ideation, the more negative social adaptation female students encounter, the more possibility of their suicidal ideation might generate. In the psychological crisis intervention on campus, setting up interpersonal communication training to improve college students’ interpersonal communication ability and social adaptability should be considered, reduce the degree of suicidal ideation, and play a positive role in suicide prevention, proving that effective offline social support may reduce psychological distress, improve coping, and reduce the potential for suicidal ideation (Luo et al., 2013; Chen and Yang, 2017).

### The direct effect of online social support among males

Contrary to our hypothesis (H1B), online social support did not directly affect the suicidal ideation of male youth. There are no relevant studies in China to support this finding, but a study by Schlichthorst et al. (2020) found that male youth with suicidal ideation reported a preference for offline social support from family, friends, or mental health professionals over online social support. Specifically, participants reported preferring to share their confusion and thoughts around suicide ideation in a more direct, face-to-face format. Meanwhile, online communication groups on suicidal issues were more commonly used by individuals in early stages of suicidal ideation or their family members and discussion centered on understanding each other’s experience; thus, such communications may not serve to prevent or reduce suicidal ideation (Schlichthorst et al., 2020). Therefore, it may be the case that online social support was not an important factor in terms of directly affecting suicidal ideation among male youth in the present study.

### The moderating effect of online social support among males

However, as hypothesized (H2B), online social support had a significant negative moderating effect on the suicidal ideation of male youth under the pressure of negative life events, such that online social support reduced the impact of all types of negative life events on suicidal ideation intensity among male youth. This finding is consistent with previous research and may be explained by the private and indirect nature of online social support. Specifically, youth experiencing suicidal ideation as a result of negative life events may turn to the Internet for support as they may be hesitant to disclose details of their negative thoughts or emotions in-person due to feelings of shame or guilt, or a desire for a more private and/or confidential social exchange that can be accomplished virtually (Liu et al., 2017). Thus, the private nature of online social support may enable youth to seek help from online forums without restraint. This online support has the potential to buffer against the negative impacts of challenging life events they may be experiencing, thereby reducing the impact of these events on their suicidal ideation intensity (Whitlock et al., 2010). In short, the inherent characteristics of online social support may be optimal for potentially reducing the impacts of negative life events on suicidal ideation among male youth.

### The direct effect of online social support among females

Contrary to our findings from male youth but consistent with our hypothesis (H1B), online social support had a significant direct effect on female youth’s suicidal ideation. Interestingly, this effect was positive, suggesting that the more online social support female youth reported, the greater the intensity of their suicidal ideation. This finding is inconsistent with previous research which has suggested that online social support may promote mental health (e.g., Schotanus-Dijkstra et al., 2014).

This may be, at least in part, explained by previous research which has found that female youth are increasingly dependent on the Internet, and may be at-risk for developing Internet addiction. In recent years, the reported rate of Internet addiction among Chinese female youth has increased. Although it is lower than that of male youth, its average annual growth rate has surpassed that of men (Zhang et al., 2017). Furthermore, Internet addiction has been found to lead to suicidal ideation and follow-up behaviors. Relevant studies have found that in the face of complex information online, Chinese female youth lacked effective self-defense awareness relative to males and were easy to be deceived (Chen et al., 2008). When female youth were exposed to an array of online social support resources, they were occasionally mixed with information regarding risky behaviors, which poses a risk of promoting suicidal ideation among female youth (Zhang et al., 2014). In short, the negative impact of online social support on female adolescents’ suicidal ideation not only came from the damage of Internet addiction to female adolescents’ learning and social ability, which made them lose confidence in real life and engage suicidal ideation afterwards, but also part of the negative information supported by the online social media engagement directly or indirectly affected the normal life of female adolescents from different angles which aggravated their suicidal ideation (Tan et al., 2017; Young et al., 2021).

### The moderating effect of online social support among females

As hypothesized (H2B), online social support had a significant negative moderating effect on the suicidal ideation of female youth under the pressure of negative life events. Thus, greater levels of online social support were found to inhibit the impact of various types of negative life events on the suicidal ideation of female youth, a finding which was similar to that from male youth and whose interpretation could be that, suicide is a controversial issue, especially for female Internet users (Li, 2020). Compared with males, female students were more interested in enhancing their psychological adjustment ability and social life adaptability *via* the internet, preventing and alleviating psychological problems, especially topics related to suicide, and helping them deal with interpersonal communication, dating, job hunting, personality development and emotional regulation (Fan, 2008). Therefore, rational use of the Internet and give full play to its positive role in regulating and moderating female students’ negative psychology.

## Limitations

The present study has following limitations: (1) The data used were from a health risk survey carried out by a research team in Shaanxi Province, China. Therefore, the sample in the present study may be considered representative of Western cities in China, but findings may not generalize to students from Eastern cities. Further studies are needed to examine the interplay between offline and online social support, negative life events, and suicidal ideation among youth in this context. (2) The present sample consisted of students attending middle high schools and universities in provincial capital and prefecture-level cities. As such, samples from county-level and township-level may not be represented within the present study. (3) This study relied only on cross-sectional self-report measures to examine suicidal ideation. Further longitudinal investigations are needed to understand potential contributors to the patterns of engagement in suicidal ideation among Chinese youth. (4) The current study is lack of the assessment of psychological disorders/suicidal ideation, models were tested in a sample of youth where the presence of psychological disorder/suicidal ideation was uncertain. Further studies are needed to investigate potential contributors to the above patterns of mechanism in suicidal ideation. (5) The sample size was unbalanced in terms of gender, results may be not reliable which should be interpreted with caution. (6) Control variables such as age, parents’ marital status were not discussed due to its subsidiarity to this research topic and the lenth of this paper, future studies are needed to better understand the impacting mechanism of these control variables to youth’s internal state of mind.

## Conclusion and implications

This is the first study to investigate the direct and moderating effects of offline and online social support on suicidal ideation among adolescents and young adults under the pressure of negative life events in Northwestern China. Our findings revealed direct and moderating effects of offline and online social support on suicidal ideation among youth under the pressure of different types of negative life events, as well as gender-specific patterns in these relationships. These findings will inform existing literature on social support, negative life events, and suicidal ideation among Chinese youth, and emphasize the need for continued efforts to explore life-threatening behaviors across various cultures and societies. Results also emphasize the need for gender-informed suicide prevention and intervention efforts among Chinese youth.

Meanwhile, the findings from the current study have important clinical implications. Results of the presented studies indicate that negative life events, offline/online social support and suicidal ideation occurs among people from non-Western cultures and in nonWestern countries but might express themselves differently. Therefore, sociocultural contexts and cultural factors relevant to each population need to be considered at both policy making and clinical setting levels to deliver culturally-tailored preventive/management strategies and treatment interventions. The acknowledgment of suicidal ideation as a clinical condition separate from suicide is the vital first step for policy makers and strategy planners. This differentiation needs to be promoted among practitioners and mental health professionals dealing with middle school and university students as well as faculty and administrative staff who may encounter such issues. The results also indicated that middle school and university mental health or counselling centers as well as websites/internet are the important resources that students seek social support from regarding their negative life events, suicidal ideation or life-threatening issues. Therefore, cultural competency among practitioners working in middle school and university mental health or counselling centers is crucial in facilitating social support seeking, disclosure of suicidal ideation in therapy, as well as maximizing positive outcomes for young students who experienced or are just about to experience suicidal ideation.

## Data availability statement

The datasets presented in this article are not readily available becauset the data that support the findings of this study are available from School of Psychology, Shaanxi Normal University, but ethical restrictions of Shaanxi Normal University apply to the availability of these data, which contains privacy variables that might affect the growth of adolescents’ mental health and were used under license for the current study, and so are not publicly available. Data are however available from the corresponding author upon reasonable request. Requests to access the datasets should be directed to xjxj4133@163.com.

## Ethics statement

The studies involving human participants were reviewed and approved by the Ethics Committee from School of Psychology, Shaanxi Normal University (Protocol Number: 18245; approved on July 12th, 2021; exempt protocol approval expiry–July 12th, 2023). Written informed consent to participate in this study was provided by the participants’ legal guardian/next of kin.

## Author contributions

MX contributed to the conceptualization of this study as well as data analysis, translation, and drafted the manuscript. JP contributed to revise and the flow of the manuscript. LZ and XY contributed to the building of macroscopical framework of this project. All authors contributed to the article and approved the submitted version.

## Funding

This study was partly sponsored by China Postdoctoral Science Foundation (CPSF), grant no. 2022M712001.

## Conflict of interest

The authors declare that the research was conducted in the absence of any commercial or financial relationships that could be construed as a potential conflict of interest.

## Publisher’s note

All claims expressed in this article are solely those of the authors and do not necessarily represent those of their affiliated organizations, or those of the publisher, the editors and the reviewers. Any product that may be evaluated in this article, or claim that may be made by its manufacturer, is not guaranteed or endorsed by the publisher.
